# Supplementary material for: Validity and intrarater reliability of a novel device for assessing Plantar flexor strength
Source: PLoS One. 2023 Mar 31;18(3):e0282395. doi: 10.1371/journal.pone.0282395 (PMC10065227; doi:10.1371/journal.pone.0282395)
Supplement: Attachment — Submitted filename: Plos One Reviewer comments and responses.docx [file pone.0282395.s001.docx]

# PLOSONE review Comments and responses

We thank the reviewers for their comments and expertise. We have individually responded to each comment giving a response and action when and where relevant. The text has subsequently been modified and additional elements are identified in yellow.

**Comment**: Reviewer #1: This is a study on the reliability and validity of a cost-effective muscle strength test device. Information on accuracy, resolution, reliability, and validity is essential in clinical practice.
In this study, the new device(hardware) whose reliability needs to be investigated is the Nintendo Wii balance board (NWBB).

Response: Regarding the hardware – this specifically includes the metal framed c station which is part of the Fysiometer system. This is critical to testing. Obviously this information was not well documented in our case so a picture has been added.

Action: we have added a picture to help show the hardware and NWBB.

**Comment**: The software - Fysiometer – does not seem to be critical. However, NWBB has already been studied in many previous studies. The differences from previous studies seemed to be clearly stated.

Response: We would argue the Fysiometer software is critical to testing as otherwise you would need to use the native Nintendo software and set up as if you were measuring an individuals weight. This would make It difficult to record peak force. As mentioned the hardware from Fysiometer is crucial to the ability to test the plantarflexors.

Action: A picture has been added to the manuscript.

**Comment** : Most previous NWBB studies investigated the inter-rater, intra-rater reliability, and concurrent validity of over 60 patients. It seems necessary to gather more subjects, and statistical methods should be reconsidered by referring to the latest similar studies.

Response: We agree, the COSMIN guidelines for reporting reliability studies were not met and we have added to the initial reliability data. We have therefore updated the paper to include a further 25 subjects who completed testing with us for test re-test reliability. Taking the size to 65. Regarding statistical methods, we are keen to keep this manuscript relevant to clinicians and therefore only reported key stats – i.e. correlation, ICC, and MDC, alongside the Bland Altman tests. If you feel we should complete some other specific tests please let us know.

Action : Manuscript modified to reflect this. But statistical tests kept the same except further information on the type of ICC completed as requested by reviewer 2.

**Comments**: In addition, the clinical applicability of the new method needs to be emphasized by highlighting its strength despite the limitation of the new device.

Response: We do not feel the new device has any real limitations other than its inability to test different contraction modes, however the sections on clinical applicability and limitations reflect this. We have modified the clinical application section to reflect your comments more specifically and emphasize the strengths of the new device in the same section and strengths/limitations section. Please also note the overall conclusion.

Actions: Modifications to the manuscript – line 262-267 and line 304-305

**Comments**: The references are old. Please add the latest studies.

Response: We would disagree with this statement. The age of a reference does not render it obsolete. References do not need to be updated unless new studies are completed – they aren’t or haven’t been and only then when they find something new. It is best to use the seminal papers in the area. This is something we have done.

If there are specific references that are outdated please do highlight them but we are not aware of any specific to the clinical side or the device.

Actions: None taken. But we are happy to amend if there are specific references identified.

**Reviewer #2: Minor comments:**
**Comments**: - Line 67-68: Add please reference to the part "(...) supporting the notion that Plantar flexor weakness may increase the risk of certain lower limb disorders.

Response: The reference at the beginning of this sentence is relevant to this statement. However for clarity we have added several to this sentence so that the audience knows it is an accepted consideration.

Action: supporting the notion that Plantar flexor weakness may increase the risk of certain lower limb disorders. (24,30)

**Comments**: - Methods: I would suggest combining Concurrent validity and Test Re-test intra-rater Reliability and creating a sub-paragraph as "Study design".
Response:

Action: We have done as suggested. Thank you for this.

**Comments:** - Line 70: Please explain the term “Gold Standard”.

Response: This terminology is a typical English statement and as such needs removing and replacing.

Action: Insertion of statement to say “which is considered the most accurate and reliable tool for measuring Plantar flexor strength”

**Major comments:**

**Comments**: - Introduction: Please add a clear perspective of the research problem, and focus on the research explanation about the novelty of this research.

Response – line 70 and 82 provides the relevant background about the problems with plantar flexor testing using IKD and leads into the development of the C-Station.

Action : We have added additional commentary into the introduction line 69-70 and 81-84 add to the existing manuscript.

**Comment**:- Introduction: Please add a hypothesis.

Response: We do not think a hypothesis is appropriate as we are examining the reliability and validity of the device. We report the study in such a way. It does not therefore warrant a hypothesis, especially as the results represent these constructs and do not test a hypothesis. The aims clearly identify the necessary information.

Action: none given

**Comment**:- Methods: An estimation of sample size and test power would be appreciated.

Response: We previously had not followed the COSMIN recommendations for sample size and have therefore modified the sample size with a further 25 subjects taking the reliability element to 65 subjects.

We have also completed an appropriate sample size calculation for an ICC/reliability study..

Action: The text has been modified to reflect the increase in sample size and a section in the method on sample size has been added.

**Comment**: - Data analysis: Please identify the exact model and type of ICC according to Koo and Li (2016).

Response: <https://pubmed.ncbi.nlm.nih.gov/27330520/> We followed the advice from the reviewer and have used the papers recommendations to form the ICC method and wording within the manuscript.

Action: Modified test (highlighted in yellow) line 189-191

**Comments**: - Results: This section should be re-arranged and focused on the exact results, including absolute and relative reliability. Currently, the section is very unclear to the reader.

Response:

Action: We have modified the results section using sub-headings to help guide the reader and modified the text. And ensured the ICC information was prominent. We feel with the addition of the tables in to the final manuscript that this section will read much more clearly.

**Comments**: - Discussion: A major comment - this section is very pour according to references and literature.

Response – It is unclear what is pour exactly in relation to the references and literature. We assume this is the lack of discussion in relation to other literature however since this is a novel test and there is no other published data we would need to discuss IKD literature. This would lose the key focus of the paper – i.e. reliability of the C station.

Action: Modifications to the Discussion . We have added more references to some statements throughout the discussion.

**Comments**: - Limitations: Please re-arrange this part.

**Comments**: Why the limitations states one page? It may indicate a huge weakness of this paper. Add the rest of this part into the discussion and prepare clear findings to discuss further.

Response: We have tried to make the limitations clear and consider what this really means in the real world by helping the reader understand the population sampled, the study design, and the positional limitations.

The size of the limitations section shows our thorough awareness of limitations rather than the condensed version requested. We feel this is better for the reader rather than limiting this discussion. Our concern with moving much of this into the discussion more generally would hinder the readers understanding and hide these issues.

We would be happy to take an editorial decision on this.

Action: the Discussion and limitations section has been altered

.
